# Cerebral Arteriovenous Malformations: Evaluation and Management

**DOI:** 10.1155/2014/649036

**Published:** 2014-10-15

**Authors:** Norman Ajiboye, Nohra Chalouhi, Robert M. Starke, Mario Zanaty, Rodney Bell

**Affiliations:** ^1^Department of Neurological Surgery, Division of Neurovascular Surgery and Endovascular Neurosurgery, Thomas Jefferson University Hospital, 901 Walnut Street, Philadelphia, PA 19107, USA; ^2^Department of Neurological Sciences, Division of Neurocritical Care, Thomas Jefferson University Hospital, Philadelphia, PA, USA; ^3^Department of Neurosurgery, University of Virginia, Charlottesville, VA, USA

## Abstract

There has been increased detection of incidental AVMs as result of the frequent use of advanced imaging techniques. The natural history of AVM is poorly understood and its management is controversial. This review provides an overview of the epidemiology, pathophysiology, natural history, clinical presentation, diagnosis, and management of AVMs. The authors discussed the imaging techniques available for detecting AVMs with regard to the advantages and disadvantages of each imaging modality. Furthermore, this review paper discusses the factors that must be considered for the most appropriate management strategy (based on the current evidence in the literature) and the risks and benefits of each management option.

## 1. Introduction

AVMs are abnormalities of the intracranial vessels that constitute a connection between the arterial and venous systems and lack an intervening capillary bed. Hemorrhagic presentation of AVM is associated with significant morbidity and mortality; it is an independent predictor of future hemorrhage. The gold standard for diagnosis of AVM is cerebral angiography. However, the evolution of noninvasive imaging techniques has led to the increased diagnosis of unruptured AVM which now presents with new challenges with regard to their management. This review paper provides a comprehensive review of the literature with presentation of the risks and benefits of the different management options available for AVM such as medical management, microsurgical resection, stereotactic radiotherapy, and endovascular embolization. Furthermore, the clinical presentation of patients with AVM and how it relates to the preferred management option are discussed in detail.

## 2. Epidemiology

Approximately 2% of AVMs are multiple and the remainder are solitary [1]. According to reports, 0.1% of the population harbors an AVM [2, 3]. Both sexes are affected equally. AVMs are the leading cause of nontraumatic intracerebral hemorrhage in people less than 35 years old [4]. Most lesions reach attention in patients in their 40's and 75% of the hemorrhagic presentations occur before the age of 50 years [2]. According to autopsy studies, only 12% of AVMs become symptomatic during life [5].

## 3. Pathology and Pathophysiology

AVMs have three components: feeding arteries, nidus, and draining veins [6]. The gross features of an AVM include the presence of single or multiple direct arteriovenous connections that permit high-flow arteriovenous shunting through small feeding arteries that lack a muscularis layer and the absence of a capillary bed. Consequently, this high-flow shunt can produce structural changes in the feeding and draining vessels which results in arterial smooth muscle hyperplasia associated with fibroblasts and connective tissue elements known as fibromuscular cushions [6]. The microscopic features of AVMs are variable and depend on the portion of the lesion that is sampled. The venous elements have thin collagenous walls and the arterial feeders have muscular elastic walls. The parenchymal elements within the AVM tend to be hemosiderin stained, gliotic, and nonfunctional. Some lesions may have vascular or interstitial calcification [7].

## 4. Natural History

AVMs are congenital lesions that occur sporadically. The familial incidence of brain AVMs is rare, and only a few reported cases in the literature; however there is an association with other abnormalities like Osler-Weber-Rendu disease and Sturge-Weber syndrome [8, 9]. Cerebral AVMs account for 1.4% to 2% of hemorrhagic strokes [10, 11]. The estimated prevalence of AVM varies from less than 10 to 18 per 100,000 [12, 13]. Brain AVMs are found incidentally on 0.05% of brain MRI screens [14].

The natural history of asymptomatic brain AVMs remains poorly understood and conflicting information can be found on symptomatic lesions in the literature. Although scientists have not reliably identified anatomic predictors, spontaneous obliteration may be more likely to occur in small AVMs (less than 2.5 cm) that present with hemorrhage and have fewer arterial feeders [15]. This phenomenon is more likely to occur with AVMs drained by a single draining vein and associated with smaller AVMs and a hemorrhagic presentation [16].

## 5. Clinical Presentation

Cerebral AVMs may present with intracranial hemorrhage, seizures, headaches, and long-term disability; the most common presenting symptoms are hemorrhage and seizures [17].

### 5.1. Intracranial Hemorrhage

The annual incidence of hemorrhage of unruptured and untreated brain AVMs is of 2% to 4% with approximately 38% to 71% of patients with brain AVMs presenting with intracranial hemorrhage [17]. There are conflicting data in regard to association of age and risk of hemorrhage; however, the initial presentation of hemorrhage most commonly occurs in patients between the ages of 20 and 40 [18, 19]. Although conflicted data exists in regard to the effect of gender on hemorrhage, evidence seems to support no effect of gender on the risk of hemorrhage [20, 21].

There is data to suggest that hemorrhagic presentation is a significant independent predictor of future hemorrhage. Ondra and colleagues [22] over a 24-year period had a prospectively followed cohort of unoperated symptomatic patients with brain AVMs and found a mean time interval of 7.7 years between initial presentation and subsequent hemorrhage. Brown Jr. and colleagues [2] found that the risk of subsequent hemorrhage fell from 32.9% during the first year to 11.3% per year in subsequent years among patients with hemorrhage as the initial presentation. A study from Toronto [17] found an annual risk of hemorrhage of 9.65% during the first year and 3.67% after 5 years from the initial hemorrhagic presentation.

Other potential risk factors for hemorrhage include (1) AVM with exclusively deep venous drainage (typically defined as drainage through the periventricular, galenic, or cerebellar pathways), (2) AVM associated with aneurysms, (3) AVMs that are deep in location, and (4) AVM that is infratentorial in location [17]. Brain AVMs are heterogeneous since each AVM's unique feature of microanatomy and vascular architecture may provide a unique clinical outcome. The risk of hemorrhage varies from 0.9% per year in patients without risk factors like hemorrhagic presentation, deep AVM location, or deep venous drainage and may be as high as 34.4% in patients with these factors [23, 24]. In a prospective study of 678 patients, the overall hemorrhage rate of brain AVMs with associated aneurysms was 6.93% per year compared with 3.99% per year for patients without associated aneurysms [17]. The risk of hemorrhage remains present until complete AVM obliteration; therefore partial endovascular embolization of an AVM does not completely reduce the risk of hemorrhage to zero [17].

### 5.2. Seizures

Seizures occur in about 18% to 40% of brain AVMs with favorable response to treatment with antiepileptic drugs [17, 25]. The type of seizure most commonly associated with AVMs is generalized seizures (30%) [26]. There is no significant association of hemorrhage with initial presentation with seizure, focal neurological deficits, or headaches as demonstrated by Brown Jr. et al. [2]. Furthermore, patients presenting with seizure are not more likely to suffer an AVM rupture during follow-up [17]. For instance, in the Toronto-based prospective study of 678 patients, the hemorrhage rate for patients presenting with seizure was 4.16% per year, which was close to the rate for the entire cohort (4.61%) [17].

### 5.3. Headache

Headaches occur in approximately 5% to 14% of patients with brain AVMs and these headaches are not distinctive. They can be unilateral or bilateral and can have migrainous features with and without aura [22, 26]. Currently, there are no studies to evaluate the rate of response to pharmacologic treatment and AVM obliteration.

### 5.4. Focal Neurologic Deficits (FNDs)

Focal neurologic deficits (FNDs) occur in 1% to 40% of patients with brain AVMs [3]. Only 5% to 15% manifest progressive deficits unrelated to hemorrhage [6]. The pathophysiology of these deficits is multifactorial and includes vascular steal phenomenon and/or venous hypertension. The vascular steal phenomenon is centered around the perinidal arterial steal, which results from the high-flow shunting through the AVM and leads to low blood pressure in the feeding arteries and surrounding brain tissue [27]. Furthermore, venous dilatation may lead to mass effect and compression of brain tissue therefore resulting in FNDs [28]. The Columbia AVM Database (with 5,735 patients) found an independent association of FND with increasing age, female gender, deep brain location, and venous ectasia without any association with lobar location, size, arterial supply, or venous drainage pattern [28].

## 6. Imaging

Conventional cerebral angiography is the gold standard in the evaluation of AVM angioarchitecture, and it shows the following essential features: the feeding arteries, location of nidus, draining veins, morphology, presence, and location of associated aneurysms, venous varices, and vasculopathic stenotic segments on arteries and veins. These features are important because they are commonly used for treatment planning [29]. One of the important angiographic features is the visualization of the AVM during the arterial phase of an early draining vein since this feature confirms the presence of an arteriovenous shunt. Intraventricular hemorrhage as a result of AVM rupture is secondary to the apex of the nidus (seen as a wedge-shaped arrangement of tangled vessels on cerebral angiography) projecting toward the ventricular surface. Furthermore, in the presence of hemorrhage, mass effect can be appreciated on an angiography. It is important to note that a thrombosed AVM may not be detected on a cerebral angiography.

Other imaging modalities which are often the initial studies used to evaluate symptoms that are not specific to AVMs include CT, CT angiography, MRI, and magnetic resonance angiography. However, these imaging techniques are limited in their sensitivity and ability to provide detailed imaging of AVM architecture [29]. Each imaging technique provides its own unique strength: CT angiography provides better vascular detail of AVMs, whereas MRI and magnetic resonance angiography provide greater visualization of surrounding structures adjacent to the nidus. Furthermore, MRI can detect thrombosed vessels as hyperintense signals and show any associated hemorrhage at various stages of evolution. T2-weighted and GRE sequences are the most sensitive to breakdown products. MRI can be important for preoperative planning because it allows for an appropriate surgical approach while demonstrating the relationship of the AVM and important parenchymal structures.

## 7. Management

An important factor that must be considered during the treatment decision-making process is to compare the risks of all treatment modalities against the natural history risks of AVMs. Management of cerebral AVMs includes observation with medical management, microsurgical resection, stereotactic radiotherapy, and endovascular embolization. Invasive treatment modalities are the reasonable choice for ruptured cerebral AVMs due to the high rate of morbidity and mortality, and the goal of treatment is eradication of the AVM. The factors that dictate treatment options (which may include single or multimodal therapy) are operator skill, AVM size and location, surgical or endovascular accessibility, venous drainage, and presence of high-risk features, such as a feeding artery aneurysm [30].

However, the appropriate treatment modalities for unruptured AVMs present a challenging clinical dilemma because of a poorly defined natural history and the seemingly low annual hemorrhage rates. This clinical conundrum led to the development of ARUBA which aims to compare the natural history with modern multimodal therapy. ARUBA (a randomized trial of unruptured brain arteriovenous malformations) was the first randomized controlled trial to evaluate surgical intervention versus medical management for unruptured cerebral AVMs. However, errors in study design, execution, short length of follow-up, and a relative lack of information regarding the treatment arm and the enrollment process invalidate the authors' conclusions [30].

### 7.1. Observation with Medical Management

Conservative treatment is considered for management of asymptomatic AVMs. Furthermore, it may include management of associated symptoms, general medical care, and surveillance imaging of an AVM. Time intervals for surveillance imaging are not well defined and may include MRI brain imaging annually or biennially. Specific medical care may include management of hypertension, headache, and seizures. Similar outcomes in seizure frequency have been reported in an observational study comparing conservative management and AVM treatment [31].

### 7.2. Microsurgical Resection

In 1986, Spetzler and Martin introduced a grading system for AVMs based on the nidus size, location in relation to eloquent cortex, and venous drainage pattern (see Table 1); site includes sensorimotor, language, visual cortex, hypothalamus, thalamus, internal capsule, brainstem, cerebellar peduncles, and cerebellar nuclei [32]. The Spetzler-Martin Scale is used to estimate the risk of surgical resection of an AVM with higher grades being associated with greater surgical morbidity and mortality [33]. Microsurgery is the gold standard for definitive treatment of AVMs. Microsurgical excision of the AVM involves a craniotomy, careful dural opening with circumferential nidus dissection until complete AVM resection is achieved. Postoperative angiography is performed to demonstrate complete AVM excision. The advantage of microsurgical resection is the high rate of complete obliteration, while the limitations of this approach include anatomic accessibility, edema from retraction, intraoperative rupture, resection of normal brain tissue, and feeding vessel thrombosis. Microsurgical adjuncts to help facilitate a better surgical outcome include the use of mapping, corticography, stimulation, and functional MRI [34]. A meta-analysis comprising 2425 patients treated between 1990 and 2000 showed a surgical mortality of 3.3% and a permanent postoperative morbidity of 8.6%, with an increasing morbidity-mortality rate associated with an increasing Spetzler-Martin grade [35].

The cure of AVM in adults is definitive with complete microsurgical resection and angiographic confirmation of obliteration. However, AVMs in children are more dynamic and may have the ability to regenerate after negative angiographic studies [36].

### 7.3. Stereotactic Radiosurgery

Radiosurgery involves the delivery of localized high-dose radiation to the AVM to induce a vascular injury, which leads to gradual sclerosis of the blood vessels with eventual obliteration over a period of 2 to 3 years. Successful treatment with radiosurgery depends on AVM size, grade, location, angioarchitecture, density of the nidus, and radiation dosage. AVMs smaller than 3.5 cm are ideal for obliteration [37]. The time from treatment to obliteration ranges from 2 to 3 years during which the patient has no protection from hemorrhage because of the delay from the effects of radiation. It was reported by Hernesniemi and colleagues [38] that the risk of hemorrhage during the initial 2 years after radiosurgery was 4.8% per year, which compares favorably with the natural history of AVMs.

However, this is less favorable with microsurgical resection. About 2 to 5 years after radiosurgery, the risk of hemorrhage is slightly higher at 5.0% per year but not significantly different from the natural history of AVMs. However, it should be noted that Pikus and colleagues demonstrated that microsurgically treated grade I to III AVMs had higher incidence of obliteration with statistically significant fewer postoperative hemorrhages, neurological deficits, and deaths when compared with stereotactic radiosurgery [39]. A multicenter analysis of 1255 patients receiving radiotherapy reported 102 (8%) patients developed a neurologic deficit after radiosurgery [40].

The advantages of radiosurgery include a noninvasive therapy and reasonable obliteration rates. The disadvantages of radiosurgery include the risk of bleeding during the latency of 1 to 2 years, neurologic deficits from edema and necrosis of normal brain tissue, and individual sensitivity to radiation and unknown long-term outcome.

### 7.4. Endovascular Embolization

Endovascular treatment of brain AVMs involves the delivery of liquid embolics, such as n-butyl cyanoacrylate and ethylene vinyl alcohol copolymer (Onyx) and platinum embolic coils via superselective catheterization with flow-guided ultrathin microcatheters. Often times, it is performed as preoperative embolization to surgical resection or in conjunction with stereotactic radiotherapy. Preoperative embolization can reduce the size of an AVM for microsurgical excision, and it has been shown to have acceptable rates of clinically significant complications (approximately 6.5%) [41].

Embolization may be performed as an adjunctive treatment to reduce the size of the AVM before radiosurgery. In certain cases, small malformations may be completely obliterated by embolization alone and large AVMs may undergo near-complete obliteration. Furthermore, palliative embolization may be used in selected cases to stabilize progression of neurologic deficits or attempt seizure control in cases where AVMs are not amenable to microsurgical excision or radiotherapy.

The advantages of endovascular therapy include a minimally invasive approach, possible immediate occlusion, and intraprocedure angiographic evaluation. The disadvantages of endovascular therapy include incomplete embolization, unintended vessel embolization, intracranial hemorrhage, and normal perfusion pressure breakthrough, leading to edema or hemorrhage [42].

## 8. Conclusion

The increasing use of advance imaging techniques will increase the incidence of asymptomatic AVMs. At the present moment, we do not fully understand the natural history of AVMs to precisely predict which AVMs will likely bleed and what the most appropriate optimal treatment option will be, single or multimodal therapy. In the future, we certainly need well designed randomized controlled trials to compare different treatment modalities and their outcomes.

## Figures and Tables

**Table 1:** Spetzler-Martin Grading Scale for AVMs.

Characteristic	Number of points assigned
Size of AVM	
Small (<3 cm)	1 point
Medium (3–6 cm)	2 points
Large (>6 cm)	3 points
Location	
Noneloquent site	0 points
Eloquent site∗	1 point
Pattern of venous drainage	
Superficial only	0 points
Deep component	1 point

∗Sensorimotor, language, visual cortex, hypothalamus, thalamus, internal capsule, brain stem, cerebellar peduncles, or cerebellar nuclei.

## Abbreviations

ARUBA: A randomized trial of unruptured brain AVMs
AVM: Arteriovenous malformation.

## References

[1] Osborn A. G., Osborn A. G. (1994). Intracranial vascular malformations. *Diagnosti Neuroradiology*.

[2] Brown R. D., Wiebers D. O., Torner J. C., O'Fallon W. M. (1996). Frequency of intracranial hemorrhage as a presenting symptom and subtype analysis: a population-based study of intracranial vascular malformations in Olmsted County, Minnesota. *Journal of Neurosurgery*.

[3] The Arteriovenous Malformation Study Group (1999). Arteriovenous malformations of the brain in adults. *The New England Journal of Medicine*.

[4] Ruíz-Sandoval J. L., Cantú C., Barinagarrementeria F. (1999). Intracerebral hemorrhage in young people: analysis of risk factors, location, causes, and prognosis. *Stroke*.

[5] McCormick W. E. (1978). Classification, pathology and natural history of angiomas of the central nervous system. *Weekly Update: Neurology and Neurosurgery*.

[6] Martin N. A., Vinters H. V., Carter L. P., Spetzler R. F., Hamilton M. G. (1995). Arteriovenous malformations. *Neurovascular Surgery*.

[7] Burger P. C., Sheithauer B. W., Vogel F. S., Burger P. C., Sheithauer B. W., Vogel F. S. (1991). Cerebrovascular disease. *Surgical Pathology of the Nervous System and Its Coverings*.

[8] Kikuchi K., Kowada M., Sasajima H. (1994). Vascular malformations of the brain in hereditary hemorrhagic telangiectasia (Rendu-Osler-Weber disease). *Surgical Neurology*.

[9] Laufer L., Cohen A. (1994). Sturge-Weber syndrome associated with a large left hemispheric arteriovenous malformation. *Pediatric Radiology*.

[10] Stapf C., Labovitz D. L., Sciacca R. R., Mast H., Mohr J. P., Sacco R. L. (2002). Incidence of adult brain arteriovenous malformation hemorrhage in a prospective population-based stroke survey. *Cerebrovascular Diseases*.

[11] Perret G., Nishioka H. (1966). Report on the cooperative study of intracranial aneurysms and subarachnoid hemorrhage. Section VI. Arteriovenous malformations. An analysis of 545 cases of cranio-cerebral arteriovenous malformations and fistulae reported to the cooperative study. *Journal of Neurosurgery*.

[12] Berman M. F., Sciacca R. R., Pile-Spellman J., Stapf C., Connolly E. S., Mohr J. P., Young W. L. (2000). The epidemiology of brain arteriovenous malformations. *Neurosurgery*.

[13] Al-Shahi R., Fang J. S. Y., Lewis S. C., Warlow C. P. (2002). Prevalence of adults with brain arteriovenous malformations: a community based study in Scotland using capture-recapture analysis. *Journal of Neurology, Neurosurgery & Psychiatry*.

[14] Morris Z., Whiteley W. N., Longstreth W. T., Weber F., Lee Y.-C., Tsushima Y., Alphs H., Ladd S. C., Warlow C., Wardlaw J. M., Al-Shahi Salman R. (2009). Incidental findings on brain magnetic resonance imaging: systematic review and meta-analysis. *The British Medical Journal*.

[15] Patel M. C., Hodgson T. J., Kemeny A. A., Forster D. M. (2001). Spontaneous obliteration of pial arteriovenous malformations: a review of 27 cases. *American Journal of Neuroradiology*.

[16] Abdulrauf S. I., Malik G. M., Awad I. A. (1999). Spontaneous angiographic obliteration of cerebral arteriovenous malformations. *Neurosurgery*.

[17] Da Costa L., Wallace M. C., Brugge K. G. T., O'Kelly C., Willinsky R. A., Tymianski M. (2009). The natural history and predictive features of hemorrhage from brain arteriovenous malformations. *Stroke*.

[18] Crawford P. M., West C. R., Chadwick D. W., Shaw M. D. M. (1986). Arteriovenous malformations of the brain: natural history in unoperated patients. *Journal of Neurology Neurosurgery and Psychiatry*.

[19] Graf C. J., Perret G. E., Torner J. C. (1983). Bleeding from cerebral arteriovenous malformations as part of their natural history. *Journal of Neurosurgery*.

[20] Mast H., Young W. L., Koennecke H.-C., Sciacca R. R., Osipov A., Pile-Spellman J., Hacein-Bey L., Duong H., Stein B. M., Mohr J. P. (1997). Risk of spontaneous haemorrhage after diagnosis of cerebral arteriovenous malformation. *The Lancet*.

[21] Stapf C., Mast H., Sciacca R. R., Choi J. H., Khaw A. V., Connolly E. S., Pile-Spellman J., Mohr J. P. (2006). Predictors of hemorrhage in patients with untreated brain arteriovenous malformation. *Neurology*.

[22] Ondra S. L., Troupp H., George E. D., Schwab K. (1990). The natural history of symptomatic arteriovenous malformations of the brain: a 24-year follow-up assessment. *Journal of Neurosurgery*.

[23] Hofmeister C., Stapf C., Hartmann A. (2000). Demographic, morphological, and clinical characteristics of 1289 patients with brain arteriovenous malformation. *Stroke*.

[24] Pollock B. E., Flickinger J. C., Lunsford L. D., Bissonette D. J., Kondziolka D. (1996). Factors that predict the bleeding risk of cerebral arteriovenous malformations. *Stroke*.

[25] Osipov A., Koennecke H.-C., Hartmann A., Young W. L., Pile-Spellman J., Hacein-Bey L., Mohr J. P., Mast H. (1997). Seizures in cerebral arteriovenous malformations: type, clinical course, and medical management. *Interventional Neuroradiology*.

[26] Fults D., Kelly D. L. (1984). Natural history of arteriovenous malformations of the brain: a clinical study. *Neurosurgery*.

[27] Mast H., Mohr J. P., Osipov A., Pile-Spellman J., Marshall R. S., Lazar R. M., Stein B. M., Young W. L. (1995). “Steal” is an unestablished mechanism for the clinical presentation of cerebral arteriovenous malformations. *Stroke*.

[28] Choi J. H., Mast H., Sciacca R. R., Hartmann A., Khaw A. V., Mohr J. P., Sacco R. L., Stapf C. (2006). Clinical outcome after first and recurrent hemorrhage in patients with untreated brain arteriovenous malformation. *Stroke*.

[29] Mossa-Basha M., Chen J., Gandhi D. (2012). Imaging of cerebral arteriovenous malformations and dural arteriovenous fistulas. *Neurosurgery Clinics of North America*.

[30] Khaw A. V., Mohr J. P., Sciacca R. R., Schumacher H. C., Hartmann A., Pile-Spellman J., Mast H., Stapf C. (2004). Association of infratentorial brain arteriovenous malformations with hemorrhage at initial presentation. *Stroke*.

[31] Josephson C. B., Bhattacharya J. J., Counsell C. E., Papanastassiou V., Ritchie V., Roberts R., Sellar R., Warlow C. P., Al-Shahi Salman R. (2012). Seizure risk with AVM treatment or conservative management: prospective, population-based study. *Neurology*.

[32] Choi J. H., Mohr J. P. (2005). Brain arteriovenous malformations in adults. *The Lancet Neurology*.

[33] Friedlander R. M. (2007). Arteriovenous malformations of the brain. *The New England Journal of Medicine*.

[34] Burchiel K. J., Clarke H., Ojemann G. A., Dacey R. G., Winn H. R. (1989). Use of stimulation mapping and corticography in the excision of arteriovenous malformations in sensorimotor and language-related neocortex. *Neurosurgery*.

[35] Castel J.-P., Kantor G. (2001). Postoperative morbidity and mortality after microsurgical exclusion of cerebral arteriovenous malformations. Current data and analysis of recent literature. *Neurochirurgie*.

[36] Turjman F., Massoud T. F., Vinuela F., Sayre J. W., Guglielmi G., Duckwiler G. (1994). Aneurysms related to cerebral arteriovenous malformations: superselective angiographic assessment in 58 patients. *The American Journal of Neuroradiology*.

[37] Friedman W. A., Bova F. J., Mendenhall W. M. (1995). Linear accelerator radiosurgery for arteriovenous malformations: the relationship of size to outcome. *Journal of Neurosurgery*.

[38] Hernesniemi J. A., Dashti R., Juvela S., Väärt K., Niemelä M., Laakso A. (2008). Natural history of brain arteriovenous malformations: a long-term follow-up study of risk of hemorrhage in 238 patients. *Neurosurgery*.

[39] Pikus H. J., Beach M. L., Harbaugh R. E. (1998). Microsurgical treatment of arteriovenous malformations: analysis and comparison with stereotactic radiosurgery. *Journal of Neurosurgery*.

[40] Flickinger J. C., Kondziolka D., Lunsford L. D., Pollock B. E., Yamamoto M., Gorman D. A., Schomberg P. J., Sneed P., Larson D., Smith V., McDermott M. W., Miyawaki L., Chilton J., Morantz R. A., Young B., Jokura H., Liscak R. (1999). A multi-institutional analysis of complication outcomes after arteriovenous malformation radiosurgery. *International Journal of Radiation Oncology Biology Physics*.

[41] Ledezma C. J., Hoh B. L., Carter B. S., Pryor J. C., Putman C. M., Ogilvy C. S. (2006). Complications of cerebral arteriovenous malformation embolization: multivariate analysis of predictive factors. *Neurosurgery*.

[42] Spetzler R. F., Wilson C. B., Weinstein P., Mehdorn M., Townsend J., Telles D. (1978). Normal perfusion pressure breakthrough theory. *Clinical Neurosurgery*.

